# Ethical problems with ethnic matching in gamete donation

**DOI:** 10.1136/medethics-2018-104894

**Published:** 2018-12-08

**Authors:** Hane Htut Maung

**Keywords:** family, minorities, political philosophy, applied and professional ethics, artificial insemination and surrogacy

## Abstract

Assisted reproduction using donor gametes is a procedure that allows those who are unable to produce their own gametes to achieve gestational parenthood. Where conception is achieved using donor sperm, the child lacks a genetic link to the intended father. Where it is achieved using a donor egg, the child lacks a genetic link to the intended mother. To address this lack of genetic kinship, some fertility clinics engage in the practice of matching the ethnicity of the gamete donor to that of the recipient parent. The intended result is for the child to have the phenotypic characteristics of the recipient parents. This paper examines the philosophical and ethical problems raised by the policy of ethnic matching in gamete donation. I consider arguments for the provision of ethnic matching based on maximising physical resemblance and fostering ethnic identity development. I then consider an argument against ethnic matching based on the charge of racialism. I conclude that while the practice of ethnic matching in gamete donation could promote positive ethnic identity development in donor-conceived children from historically subjugated ethnic minorities, it also risks endorsing the problematic societal attitudes and assumptions regarding ethnicity that enabled such subjugation in the first place.

## Introduction

For people who wish to have children but are unable to produce their own gametes, assisted reproductive procedures involving gametes from donors offer ways of becoming gestational and social parents. A result of conceiving with donor gametes is that the children will lack genetic ties with one or both of the parents. In order to compensate for this lack of genetic kinship, some fertility clinics engage in the practice of matching gamete donors with recipient parents with respect to ethnicity. The purpose is to increase likelihood that the resulting child will have the phenotypic characteristics of the recipient parent despite the absence of a direct genetic link.

In the UK, the matching of gamete donors and recipient parents with respect to ethnicity used to be official policy. The sixth edition of the Human Fertilisation and Embryology Authority’s (HFEA) *Code of Practice* from 2003 states:

Where treatment is provided for a man and woman together, treatment centres are expected to strive as far as possible to match the physical characteristics and ethnic background of the donor to those of the infertile partner, or in the case of embryo donation, to both partners, unless there are good reasons for departing from this procedure.^1^

However, there has since been a sharp change in the HFEA’s policy. The revised eighth edition of its *Code of Practice* from 2014 states:

Centres are not expected to match the ethnic background of the recipient to that of the donor. Where a prospective recipient is happy to accept a donor from a different ethnic background, the centre can offer treatment, subject to the normal welfare of the child assessment.^2^

In spite of this change in HFEA policy, individual clinics in the UK continue to offer ethnic matching of gamete donors and recipient parents. Ethnic matching in gamete donation has also been a standard practice in other countries, including Spain, Norway, Finland and the USA.^3–5^

In this paper, I expose and evaluate the philosophical and ethical issues raised by the policy of ethnic matching in gamete donation. I consider arguments for the provision of ethnic matching that appeal to the parental wishes to maximise the resemblance between the parents and child and to the fostering of the child’s ethnic identity. I then consider an argument against ethnic matching that appeals to the charge of racialism. I conclude that the provision of ethnic matching in gamete donation is a double-edged sword. While the practice could promote positive ethnic identities in subjugated ethnic minorities, it also presupposes the problematic societal attitudes and assumptions concerning ethnic categories that enabled such subjugation in the first place.

Before I proceed, two clarifications are in order. First, the present paper is predominantly concerned with the ethics of the policy of offering ethnic matching in gamete donation, rather than the ethics of the individual’s choice to request ethnically matched donor gametes. That is to say, my aim is to expose the ethical issues that arise from having a reproductive donation policy that offers ethnic matching, but not to evaluate whether or not any given individual’s request for ethnically matched donor gametes is justified. The two topics are clearly connected, but it is important to distinguish them, because a policy influences societal attitudes and the aggregate consequences of individual choices far more than any given individual choice does. Hence, there are considerations which may be more pertinent to the ethics of the policy of ethnic matching than to the ethics of the individual choice to use ethnically matched donor gametes.

Second, the present paper uses the term ‘ethnic’ rather than ‘racial’ matching in order to keep with the language used in the HFEA’s policy documents, which explicitly refer to matching based on ‘ethnic background’.^1 2^ As noted by Cornell and Hartmann, the two concepts are discursively linked, but ‘race’ is usually taken to be a phenotypic classification based on ‘perceived common physical characteristics that are held to be inherent’, while ‘ethnicity’ is usually taken to be a cultural and historical grouping based on ‘past linguistic heritage, religious affiliations, claimed kinship, or some physical traits’.^6^ However, the precise boundary between ‘race’ and ‘ethnicity’ is contested, with the two concepts being conflated or the latter being thought to subsume the former. The HFEA’s language may be supported by the fact that the characteristics on which such matching is based include not only phenotypic traits, but also claimed heritage and kinship, which are typically associated with ‘ethnicity’. Nonetheless, given that such ‘ethnic’ matching also involves the classification and matching of people based on characteristics that are typically associated with ‘race’, such as skin colour and hair colour, it raises ethical concerns regarding racialism and racism.

## Family resemblance

A major motivation for the ethnic matching of a gamete donor and a recipient parent is that it maximises the chances of the donor-conceived child resembling his or her parents. Such resemblance may be valued because it allows the child to be seen by others to ‘fit in’ with the family. As Pennings notes, the practice of matching reflects a desire ‘to conform the new-founded family to the ideal of the natural family’.^7^ Furthermore, as Quiroga notes, this allows the family to ‘maintain secrecy about the use of a donor by ensuring that the child could ‘pass’ as a genetic child’.^5^ The argument, then, is that the policy of ethnic matching is justified because it allows the family the opportunity to give the public appearance of being a ‘normal’ genetically related family whose members resemble each other. However, this line of argument is liable to some objections.

The first objection is that the emphasis on the public appearance of the family undermines what McDougall calls the parental virtue of acceptance, according to which parents ought to accept their child regardless of his or her specific characteristics.^8^ However, the practice of ethnic matching seems to suggest that the parents’ willingness to accept a child is conditional on the prospect of their fulfilling the desire to give the public appearance of being a certain kind of family. Therefore, the parental desire to maximise resemblance fails to provide an ethical justification for a reproductive donation policy that offers ethnic matching.

Another way to couch this objection is to say that the choice to reject a potential child on the basis of a potential lack of resemblance is the result of what Asch and Wasserman call synecdoche, which is ‘the uncritical reliance on a stigma-driven inference from a single feature to a whole future life’.^9^ Such a synecdochal choice reduces the value of the potential child to a single characteristic, such as resemblance, and assumes that the potential child, in virtue of this characteristic, would not be able to satisfy the goods of family life. The practice of ethnic matching for the purpose of maximising resemblance, insofar as it amounts to selecting potential children on the grounds of whether they would be likely to be seen to ‘fit in’ with their prospective families, encourages such synecdochal attitudes.

In response, it could be countered that the parental virtue of acceptance is too general, as it would also condemn all kinds of prenatal selection and matching. Indeed, scholars have appealed to the parental virtue of acceptance and synecdoche to criticise prenatal sex selection and selection against disability.^8 9^ The parental virtue of acceptance could also suggest that conceiving a genetically related child is no more preferable to donor conception or adoption, insofar as the acceptance of a child should not conditional on whether or not the child is genetically related to the parents. Hence, the objection based on the parental virtue of acceptance and synecdoche does not seem to pose a specific problem for ethnic matching.

Nonetheless, there is a reason why the objection based on the parental virtue of acceptance and synecdoche is poignant in the case of ethnic matching. As suggested by Asch and Wasserman, ‘the actual or hypothetical refusal to enter into an intimate relationship with someone is not consistent with regarding that person, or people like him in relevant respects, as an equal if the characteristic on which the refusal is based is integral to the person’s identity, or if it has been subject to a history of persecution and stigmatisation’.^9^ The preference for a potential child who publicly resembles the recipient parent with respect to ethnicity reflects the judgement that ethnicity is relevant to the child’s ability to satisfy the goods of family life, which is especially problematic because ethnicity is a feature of a person’s identity which has been the basis for persecution and stigmatisation.

This links in with the second objection to ethnic matching for the purpose of maximising resemblance, which is that the practice ratifies and perpetuates problematic societal attitudes regarding physical appearance and what constitutes a ‘normal’ family. A great deal of value is placed on resemblance as a mark of the ‘normal’ family in our society and this is often uncritically assumed to be biologically grounded.^10 11^ What this fails to recognise, though, is the extent to which the value we place on resemblance is culturally contingent. For example, Roberts notes that families in the African-American community do not place such value on resemblance, because they acknowledge the diversity of genetic backgrounds within their community.^12^ The value we place on resemblance, then, is not universal, but is informed by a normative conception of family that is particular to a given cultural standpoint.

As Witt and Haslanger note, judgements about whether family members resemble each other are not straightforwardly descriptive, but are influenced by societal norms and assumptions that govern what kinds of resemblance are salient to us.^10 13^ While there are many ways in which a child can resemble his or her parents, we only consider some of these ways to be relevant as markers of genetic relatedness. In the particular normative conception of family prevalent in the USA and parts of Europe, resemblances based on characteristics that are linked to race and ethnicity are considered especially salient. For example, Rulli notes that our particular normative conception of family ‘can accommodate differences in gender (a girl can resemble her father) but not differences in race (a black child does not so easily resemble her white mom in the relevant way, even though there may be physical similarities)’.^11^ Similarly, Quiroga notes this normative conception of family which considers racialised traits to be salient rests on the assumption that ‘(p)arents and children must have a phenotypic resemblance (similar physical features) so all family members must be of the same race’.^5^

And so, the policy of ethnic matching for the purpose of maximising resemblance is situated in the context of a particular normative conception of family that takes resemblances based on racialised traits to be salient. Uncritically, promoting this normative conception of family risks the ‘othering’ of families who do not conform to this conception as being illegitimate or inferior, including families with adopted or donor-conceived children who do not share the ethnic backgrounds of their parents. That is to say, the significance placed on ethnic matching reflects the assumption that ‘normal’ families are families whose members resemble each other in certain ways, which implies that families whose members do not resemble each other in these ways are not ‘normal’ families. Also, in virtue of the emphasis that this normative conception of family places on racialised characteristics, promoting this normative conception of family can involve racialist thinking. I reserve a more detailed discussion of this point for later.

## Ethnic identity

The above considerations provide some reason to be suspicious of the parental desire to maximise resemblance as a justification for the policy of ethnic matching in gamete donation. The same criticisms also apply to the parental wish for secrecy, insofar as this wish reflects a desire for the future child to publicly ‘pass as a genetic child’ according to certain presumed markers of resemblance that are deemed salient by a particular normative conception of family.^5^ Nonetheless, I propose that there is another important rationale for ethnic matching, which is to allow the resulting child to grow up in a family environment that can support the development of a positive ethnic identity. This line of argument has been used to support ethnic matching of children and prospective parents in the context of adoption, but I suggest that it can also be used to support ethnic matching in the context of gamete donation.^14^

The development of a positive ethnic identity is considered especially crucial where the child is from a historically subjugated ethnic minority. The argument is that ethnic matching would allow a donor-conceived child from an ethnic minority to grow up and bond with a family and community of people who have the same ethnic background, which would enable him or her to experience value and meaning in belonging to a community with a shared kinship and cultural history. Furthermore, a positive ethnic identity, combined with the support from family members who may have themselves experienced discrimination because of their ethnicity, could endow the child with the resilience and resources to resist the racism he or she might experience.^15^ By contrast, such a context for developing a positive ethnic identity and resilience to racism may not be available to a donor-conceived child of mixed or minority ethnicity born to parents from a socially privileged ethnic majority who are unfamiliar with experiences of racism.

The argument from ethnic identity is commendable for placing the social and developmental needs of donor-conceived children from ethnic minorities at the forefront of the discussion about ethnic matching, but there are some potential objections that must be addressed. First, it could be objected that our duties concerning the social and developmental needs of donor-conceived children only apply to actual children, but not to potential children. Given that ethnic matching in gamete donation is a practice that pertains to potential children who have not yet been conceived, it could be claimed that the argument from ethnic identity is not relevant. However, I argue that this objection does not hold. If the needs of future potential children are irrelevant, then all of our policies that are motivated by considerations about future generations would also be unjustified. These include strategies relating to preconception public health and to environmental sustainability, such as preconception rubella vaccination programmes and renewable energy infrastructure investment, respectively. Accordingly, considerations about the social and developmental needs of potential children conceived through donor gametes are not irrelevant when devising a policy of gamete donation.

Second, given that the argument from ethnic identity is an argument based on the welfare of the potential child, it raises the question of whether being ethnically matched to the recipient parents is in the best interests of the potential child. For example, Savulescu and Kahane suggest that solely with respect to the concern for the welfare of the future child, it might be preferable for a recipient parent from a minority or mixed ethnic background to use gametes from a white European donor, because having lighter skin would prevent the potential child from being subject to racism.^16^ However, a serious problem with this suggestion, noted by Sparrow, is that the practice of selecting for lighter skin is complicit with racist oppression.^17^ While selecting for lighter skin might protect the individual child from racism, it capitulates to the unjust social order that disadvantages people with dark skin in the first place. Such capitulation is inconsistent with regarding people from ethnic groups with dark skin as equals. Accordingly, Savulescu and Kahane concede that selecting for lighter skin is objectionable because the concern for the welfare of the potential child is outweighed by the concern about colluding with racism.

The policy of ethnic matching for the purpose of promoting positive ethnic identity development in donor-conceived children does not have the obvious racist implications of the practice of selecting for lighter skin. Therefore, in virtue of the concern about capitulating to an unjust social order and in virtue of the potential benefits of positive ethnic identity development, ethnic matching is far more acceptable than selecting for lighter skin. However, this is not to say that ethnic matching avoids this concern altogether. While it may avoid the direct racist implications of selecting for lighter skin, I argue that the policy of ethnic matching, nonetheless, presupposes and potentially perpetuates racialist thinking. I now turn to this charge of racialism.

## Racialism and racism

Appiah characterises racialism as the doctrine that ‘there are heritable characteristics, possessed by members of our species, which allow us to divide them into a small set of races, in such a way that all the members of these races share certain traits and tendencies with each other that they do not share with members of any other race’.^18^ Racialism can be distinguished from racism, which broadly refers to the prejudice against a person or a group of people based on assumptions about their race. Nonetheless, the two concepts are closely linked. Although racialism does not entail racism, racism presupposes racialism. Hence, as we shall see, racialism can enable racism.

As noted by Fogg Davis, ethnic matching in gamete donation presupposes racialism by suggesting ‘that a person’s gametes are transmitters of racial meaning that can and should be selectively transmitted to their child through the use of reproductive technologies’.^19^ We saw earlier that the normative conception of family prevalent in the USA and parts of Europe places a great deal of emphasis on resemblances based on traits that are considered to be markers of ethnicity and that are assumed to be genetically inherited. The practice of ethnic matching involves classifying gamete donors and recipient parents into ethnic categories, and then matching them based on these categories, in order to produce children who have the ethnic properties of their gamete donors and hence of their recipient parents. This assumes a form of biological essentialism, whereby (1) the gamete donor and the recipient parent belong to the same ethnic category in virtue of their sharing some biologically significant ethnic property or property cluster, (2) the donor-conceived child inherits this ethnic property or property cluster from the gamete donor and (3) these conditions result in the donor-conceived child also sharing this ethnic property or property cluster with the recipient parent.

Such essentialism is problematic for two reasons. The first reason is that it lacks scientific credibility. The discursive linking of ethnic classification and biological essentialism has a long history, much of which is associated with the attempt to vindicate the ideology of colonial imperialism, but essentialism has since been discredited.^18^ Zack provides a comprehensive summary of the evidence against racialism, including the failure of racial and ethnic categories to correspond to genetic essences, the greater degrees of genetic variation within ethnic groups than between them and the roles of geographically based environmental stimuli in continuously shaping phenotypic traits.^20^ Rather than reflecting biological categories, some philosophers propose that racial and ethnic categories are social categories that are genetically insignificant.^21 22^ Therefore, insofar as it erroneously reifies ethnic categories as biological categories, the policy of ethnic matching could perpetuate misunderstandings about what properties are inherited through our genes and what our ethnic categories really reflect.

The second reason why racialism is problematic is that it enables stereotyping and racist oppression. This is because ethnic categories are often not purely descriptive, but normative.^22^ The putative biological essences that purportedly determine ethnicity are considered to explain physiological and psychological differences between people from different ethnic groups. This encourages stereotyping and constrains individual autonomy by prescribing to members of an ethnic group certain scripts or ways of acting that are considered typical to that ethnic group in virtue of intrinsic biological essences.^23^ The concern about racism arises when certain physiological or psychological traits are valued more than others are. The assumption that certain valued traits are the products of the intrinsic biological properties of particular ethnic groups easily risks slipping into the attitude that some ethnic groups have features that are ‘superior’ to those of others, or even the attitude that some ethnic groups are intrinsically ‘inferior’ to others.^24^

In the context of gamete donation, these attitudes can manifest in the desires of prospective parents from a socially privileged ethnic majority to reproduce in their donor-conceived children certain valued characteristics they associate with their ethnic group. Homanen presents evidence of such desires in her study of fertility clinics in Finland.^4^ She gives examples of white recipient parents from Finland, Norway and Sweden who value the ethnic matching of gamete donors because it allows their children to inherit the white ‘Nordic look’ and enables ‘Nordicness’ to be conserved in their families. Furthermore, the fertility clinic websites reinforce these attitudes by posting photographs of babies and donors with fair phenotypic features and by stating that recipients will be provided ‘with gametes of domestic origin’. Therefore, a worry about the policy of ethnic matching in gamete donation is that it can play into the dubious incentive of maintaining the ‘purity’ of the socially privileged ethnic majority.

The concerns about racialism seem to support a move away from ethnic matching and towards a colour-blind policy in gamete donation. If fertility clinics refuse to engage in the ethnic matching of gamete donors and recipient parents, then they avoid promoting erroneous ideas about the biological significance and genetic transmission of ethnicity. This could help to curtail harmful stereotyping and discrimination based on ethnicity, which in turn could encourage the egalitarian view that differences in skin colour, hair colour and ancestry do not matter in the context of forming a loving family.

However, such a colour-blind policy is problematic, because it overlooks the institutional structures and implicit cultural conventions in our society that selectively privilege people from the dominant ethnic majority and disadvantage those from historically subjugated ethnic minorities. To ignore differences in ethnicity is to ignore the harms that disproportionately affect people from minority ethnic groups due to these structures and conventions.^25 26^ Therefore, there remains a need to acknowledge different ethnic identities if we want to avoid perpetuating injustice. Nonetheless, this does not require a return to racialist thinking, as it is entirely reasonable to hold the view that ethnic identities are social categories while recognising that the members of these social categories suffer disproportionate harms.^24^ Such a need to acknowledge different ethnic identities provides a reason to support ethnic matching in gamete donation, especially with respect to addressing the challenges faced by people from historically subjugated ethnic minorities. In addition to the argument based on welfare presented in the previous section, offering ethnic matching to prospective parents from marginalised ethnic minorities could be seen as respecting the rights of these marginalised communities to maintain their ethnic identities.

## Conclusion

The ethical terrain surrounding the policy of ethnic matching in gamete donation is marked by tensions between the concern for the prospective parents’ wishes, the concern for the welfare of the future child and the concern about complying with racialism. I have argued that the parental desire for family resemblance fails to provide a satisfactory justification for ethnic matching because it promotes a particular normative conception of family that places undue emphasis on resemblances based on racialised traits. An ethnic matching policy might be justified on the basis of concern for the welfare of the future child, because it could allow a donor-conceived child from a historically subjugated ethnic minority to develop a positive ethnic identity and to experience meaning in belonging to a community with a shared cultural history. However, this concern for the welfare of the future child is counterbalanced by the concern about capitulating to the racialist assumptions that had enabled such subjugation in the first place. As suggested by Homanen’s study, there is a worry that a policy of ethnic matching could play into a desire to conserve certain traits that are judged to be ‘superior’ due to their being associated with the socially privileged ethnic majority.^4^ Nonetheless, offering ethnic matching to prospective parents from marginalised ethnic minorities could also be seen as respecting the rights of these marginalised communities to maintain their ethnic identities. This suggests the need for policymakers to keep considerations about the social challenges faced by less privileged ethnic minority communities at the forefront when deciding whether or not to endorse a policy of ethnic matching in gamete donation.
